# Pulmonary cavitations with increased ^18^F-FDG uptake revealing a thoracic endometriosis: A case report

**DOI:** 10.1097/MD.0000000000027550

**Published:** 2021-10-22

**Authors:** Pauline Mulette, Amaury Jacquet, Anne Durlach, Dimitri Papathanassiou, Paul Lalire, Olivier Graesslin, Gonzague Delepine, Sandra Dury, Valérian Dormoy, Jeanne-Marie Perotin, François Lebargy, Gaëtan Deslée, Claire Launois

**Affiliations:** a Department of Respiratory Diseases, Reims University Hospital, France; b Department of Pathology, Reims University Hospital, France; c Pulmonary Pathologies and Cellular Plasticity, Inserm UMR-S 1250, Reims, France; d Department of Nuclear Medicine, Jean Godinot Institute, Reims, France; e Laboratory of Biophysics, Research Unit of Medicine, University of Reims Champagne-Ardenne, Reims, France; f Science and Information Technology Research Center (CReSTIC) EA 3804, University of Reims Champagne-Ardenne, Reims, France; g Department of Obstetrics and Gynecology, Reims University Hospital, France; h Department of Cardiothoracic Surgery, Reims University Hospital, France.

**Keywords:** [^18^F] fluoro-2-deoxy-D-glucose, case report, computed tomography, positon emission tomography–computed tomography, thoracic endometriosis

## Abstract

**Rationale:**

Thoracic endometriosis is a rare disorder that can involve airways, pleura and lung parenchyma. It is the most frequent form of extra-abdominopelvic endometriosis. Multiple lung cavitations are a rare feature of thoracic endometriosis.

**Patient concerns:**

A 46-year-old woman was referred to our hospital after incidental finding of multiple pulmonary cavitations with surrounding areas of ground glass opacity on a thoraco-abdominal computed tomography-scan performed for abdominal pain. Retrospectively, the patient also reported mild hemoptysis occurring 4 months ago.

**Diagnoses:**

Positron emission tomography–computed tomography scan revealed moderate and homogeneous [^18^F] fluoro-2-deoxy-D-glucose (^18^F-FDG) uptake in pulmonary cavitations (maximum standardized uptake value 5.7). The diagnosis of thoracic endometriosis was confirmed by histological examination of surgical resection of a left lower lobe cavitation.

**Interventions and outcome:**

Gonadotropin-releasing hormone analogues associated with add-back therapy was started. Four months after initiating pharmacological treatment, the chest computed tomography-scan showed a dramatic decrease in lung cavitations size.

**Lessons:**

Thoracic endometriosis is a rare disorder requiring a multidisciplinary management including gynaecologist, pulmonologist, radiologist, nuclear physician, pathologist and thoracic surgeon for early diagnosis and treatment. Our case report highlights that an increased ^18^F-FDG uptake can be found in thoracic endometriosis syndrome presenting as multiple lung cavitations.

## 1 Introduction

Endometriosis is an estrogen-dependent condition characterized by endometrial tissue present outside the uterine cavity. It is frequently associated with inflammation, chronic pain and infertility.^[1]^ The pelvis is the main localization of endometriosis, however it can also be found in the gastrointestinal tract, the urinary tract, the lung, the diaphragm, the pericardium, the central nervous system or the skin.^[2]^ Endometriosis affects approximately 6% to 10% of women in reproductive age.^[3]^ Thoracic endometriosis syndrome (TES), described for the first time by Schwarz in 1938, is defined by the presence of ectopic intrathoracic endometrial tissue and is the most frequent extra-abdominopelvic site of endometriosis.^[4]^ TES is rare and the diagnosis is often delayed because of poor awareness. TES includes a spectrum of clinical and radiological thoracic manifestations, including catamenial pneumothorax (73% of TES cases), catamenial hemothorax (14%), hemoptysis (7%) and pulmonary nodules (6%).^[5]^ TES is not systematically associated with pelvic endometriosis. In 2 large retrospective studies, the mean age at the onset of TES symptoms was 35 years old.^[5,6]^ Chest radiography, chest computed tomography (CT-scan), and magnetic resonance imaging are useful in evaluating patients presenting with pneumothorax, hemothorax, hemoptysis, or lung nodules. A few data are available regarding the interest of [^18^F] fluoro-2-deoxy-D-glucose (^18^F-FDG) positron emission tomography–computed tomography (PET–CT) in the diagnostic approach of TES.

## 2 Case presentation

**Figure 1:**
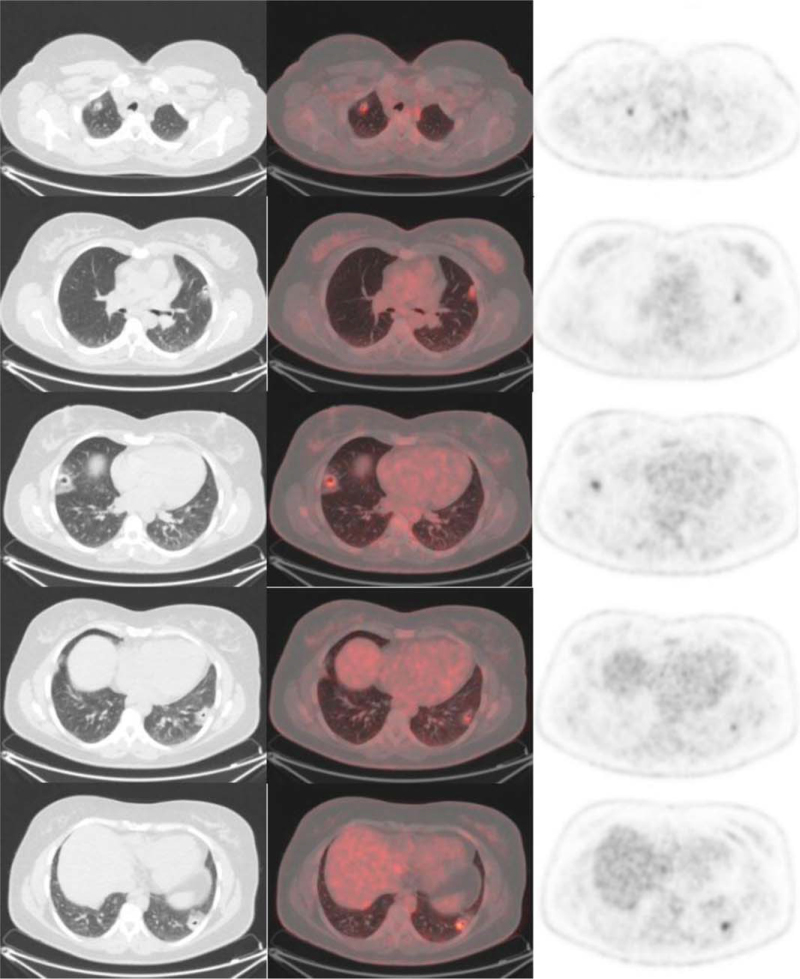
^18^F-FDG PET–CT scan findings. Transverse CT (left column) with corresponding transverse fusion image (middle column) and corresponding PET (right column) which showed pulmonary cavitations with surrounding ground glass at different level. From top to bottom, the SUV max value was 4,1; 3,9; 5,3; 3,4; and 5,7. ^18^F-FDG = [^18^F] fluoro-2-deoxy-D-glucose, PET–CT = positon emission tomography–computed tomography, SUV = standardized uptake value.

**Figure 2:**
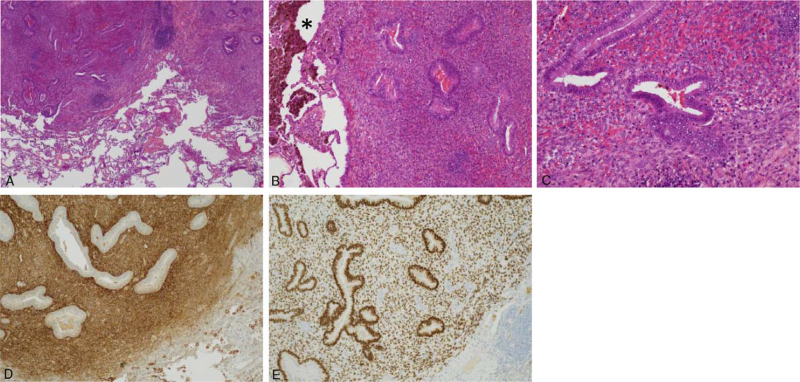
Pathological examinations of the lower lung cavitation. Hematoxylin-eosin-saffron (HES) stained at ×40 (A), ×100 (B), and ×200 (C) magnification showing endometrial glands, endometriotic stroma and hemosiderin-laden macrophages^∗^. Immunohistochemical staining of CD10 showing immunoreactivity for endometrial stroma at ×100 (D). Immunohistochemical staining of estrogen receptor showing nuclear immunoreactivity for both endometrial stroma and gland at ×100 (E).

A 46-year-old woman was referred to our hospital in October 2018 after incidental finding of multiple pulmonary cavitations on abdominal CT-scan performed in a context of chronic abdominal pain in June 2018. The presence of these bilateral cavitations with surrounding ground-glass was confirmed by chest CT-scan in June and August 2018. No prior thoracic imaging was available and the age of cavitations could then not be established. The patient did not describe any respiratory or thoracic symptoms except for a unique episode of mild hemoptysis in June 2018. There was a history of former smoking which was stopped in 2004, estimated at 1.5 pack-year. Fiberoptic bronchoscopy showed the absence of endobronchial abnormalities with no bleeding. The results of bronchoalveolar lavage fluid tests for common bacteria, tuberculosis, and fungi were all negative. Cytologic analysis of bronchoalveolar lavage identified 228,000 cells/mL, including 90% macrophages, 8% lymphocytes, and 2% neutrophils with no malignant cells; the Golde score was 319, suggesting intra-alveolar hemorrhage. Laboratory findings did not reveal any argument for an autoimmune disease, parasitosis or pulmonary aspergillosis. Cardiac ultrasound was normal. In November 2018, a PET–CT (GE Discovery 710) was performed after 6 hours of fasting. PET–CT images were acquired from vertex to mid-thigh 1 hour after intravenous injection of ^18^F-FDG (3 MBq/kg) and revealed moderate and homogeneous FDG uptake in lung cavitations, with the maximum standardized uptake value found in a left lower lobe cavitation (maximum standardized uptake value 5.7). The PET–CT also confirmed the presence of a left anterior pelvic formation without significant hypermetabolism, as described in the abdominal CT-scan in June 2018 and no other significant FDG uptake (Fig. 1). Because of diagnostic uncertainty, the patient underwent a surgical resection of the most hyperfixating left lower lobe cavitation by video-thoracoscopy in February 2019. Histopathological examination on formalin-fixed paraffin-embedded tissues revealed ectopic endometrial implants containing both endometriotic glands and endometrial stroma with positive immunostaining for estrogen receptor and CD10 (Fig. 2).

Regarding gynecological history, the patient described cyclic abdominal pain evolving for years and postpartum hemorrhage in October 2007 after the second caesarean requiring subtotal hysterectomy and right annexectomy with no histologic lesion of endometriosis. Pelvic magnetic resonance imaging showed thickening of the utero-sacral ligaments, the torus uterinum and the left round ligament with endometriotic implants in right pelvic muscles, indicating moderate lesions of pelvic endometriosis.

After multidisciplinary discussion, gonadotropin-releasing hormone analogues (Triptorelin 11.25 1 injection every 3 months) associated with add-back therapy (transdermal estrogen therapy) was initiated 7 months after the diagnosis. After 2 injections, a chest CT-scan was performed and identified an overall decrease in the size of cavitations and the absence of new cavitations. One year after pharmacological treatment initiation, 5 episodes of mild hemoptysis with no periodicity were noted and thoracic CT-scan showed stability in size and number of pulmonary cavitations with surrounding ground-glass. The pharmacological treatment was maintained. Follow-up visits and CT-scan until February 2021, confirmed the stability of lung cavitations with rare episodes of mild hemoptysis. The patient was satisfied with this effective treatment for chronic abdominal pain.

Written informed consent was obtained from the patient for publication of the case details and accompanying images.

## 3 Discussion

Multiple lung nodules and cavitations are a rare presentation of TES, leading to frequently delayed diagnosis because of relatively poor awareness. By contrast with other entities of TES occurring more frequently in younger patients, lung nodules and cavitations are more frequent in women over 35 years.^[5,6]^ Lung nodules in TES can be asymptomatic or associated with cough, dyspnea, or hemoptysis as in our case report. The size of lung nodules usually varies from 0.5 to 3 cm^[5]^ and can be associated with cavities as in our case report. Classic histopathological features of endometriosis include endometrial glands, stroma, and hemosiderin-laden macrophages. However, these elements are not always identified, therefore the histologic diagnosis can be challenging on small tissues specimen from pleural or bronchial biopsies.^[7]^ The gold standard for a definitive diagnosis of TES is histologic analysis of samples obtained from video-assisted thoracoscopic surgery.^[8]^

The most important finding of our observation is the moderate and homogeneous FDG uptake found in PET–CT can. These PET–CT scan findings are usually associated with neoplasia, infectious or inflammatory etiologies. Our case report highlights that FDG metabolic activity can occur in the presence of thoracic endometriosis. Of note, in a prospective study assessing 10 patients with suspected abdominopelvic endometriosis, none of the patients exhibited FDG hypermetabolism on PET–CT,^[9]^ suggesting no hypermetabolic activity associated with endometriosis. However, a significant FDG uptake has been reported in pelvic endometriosis in 2 case reports.^[10,11]^ To our knowledge, only 1 other case report described an intense FDG uptake in a cavitary lung mass on PET–CT in a 47-year-old woman with a final diagnosis of endometriosis.^[12]^ The FDG metabolic activity in TES presenting as lung cavitations might be related to endometriosis, but also to loco-regional inflammation with macrophages associated with the excavation of the lung nodules.

Therapeutic management of TES includes simple observation, medical treatment, surgery or combination of therapy. The first-choice pharmacological treatments are gonadotropin-releasing hormone analogs (GnRh analogs) aiming to suppress the hypophyseal-gonadal axis with the goal of suppressing ovarian steroid hormone production in order to inhibit lesion growth.^[8,13]^ In multiple lung nodules, medical treatment is the first-choice. Our case report highlights the efficiency of gonadotropin-releasing hormone analogues with a dramatic decrease in lung cavitations size. Few studies assessed the evolution of TES. In a retrospective study, including 15 patients with TES (8 patients with catamenial hemoptysis and 7 with catamenial pneumothorax), 33% had clinical signs of recurrence during the follow-up. Of note, recurrence was not detected in any of 5 catamenial pneumothorax patients treated by adjuvant hormonal therapy after surgery. Combination therapy may be the preferred approach to prevent long-term morbidity.^[14]^

## 4 Conclusion

TES is a rare disorder requiring a multidisciplinary management including gynaecologist, pulmonologist, radiologist, nuclear physician, pathologist, and thoracic surgeon for early diagnosis and treatment. Our case report highlights that an increased ^18^F-FDG uptake can be found in TES presenting as multiple lung cavitations.

## Author contributions

**Conceptualization:** Pauline Mulette, Gaëtan Deslée, Dimitri Papathanassiou.

**Validation:** Gaëtan Deslée, Claire Launois.

**Writing – original draft:** Pauline Mulette, Gaëtan Deslée.

**Writing – review & editing:** Pauline Mulette, Anne Durlach, Amaury Jacquet, Dimitri Papathanassiou, Paul Lalire, Olivier Graesslin, Gonzague Delepine, Sandra Dury, Valérian Dormoy, Jeanne-Marie Perotin, François Lebargy, Gaëtan Deslée, Claire Launois.
